# Comparing transfusion reactions between pre-storage and post-storage leukoreduced apheresis platelets: an analysis using propensity score matching

**DOI:** 10.1007/s00277-024-05652-9

**Published:** 2024-02-23

**Authors:** Sheng-Hsuan Chien, Hsin-Yi Huang, Ying-Ju Chen, Yu-Chen Tsai, Shu-Hua Lu, Li-Hsuan Lee, Hsueng-Mei Liu, Wen-Chun Chen, Yao-Chung Liu, Ting-An Lin, Chun-Yu Liu

**Affiliations:** 1 Division of Transfusion Medicine, Department of Medicine, Taipei Veterans General Hospital, No. 201, Sec 2, Shih-Pai Road, Taipei, 112 Taiwan; 2 Faculty of Medicine, National Yang-Ming Chiao Tung University, Taipei, 112 Taiwan; 3 Institute of Clinical Medicine, National Yang-Ming Chiao Tung University, Taipei, 112 Taiwan; 4 Biostatistics Task Force, Taipei Veterans General Hospital, Taipei, 112 Taiwan; 5 Division of Hematology, Department of Medicine, Taipei Veterans General Hospital, Taipei, 112 Taiwan

**Keywords:** Transfusion reaction, Apheresis platelets, Leukoreduction, Pre-storage, Post-storage, Propensity score

## Abstract

Transfusion reactions induced by platelet transfusions may be reduced and alleviated by leukocyte reduction of platelets. Although leukoreduction of apheresis platelets can be performed either pre-storage or post-storage, seldom studies directly compare the incidence of transfusion reaction in these two different blood products. We conducted a retrospective study to compare the transfusion reactions between pre-storage and post-storage leukoreduced apheresis platelets. We reviewed the general characteristics and the transfusion reactions, symptoms, and categories for inpatients who received pre-storage or post-storage leukoreduced apheresis platelets. Propensity-score matching was performed to adjust for baseline differences between groups. A total of 40,837 leukoreduction apheresis platelet orders were reviewed. 116 (0.53%) transfusion reactions were reported in 21,884 transfusions with pre-storage leukoreduction, and 174 (0.91%) reactions were reported in 18,953 transfusions with post-storage leukoreduction. Before propensity-score matching, the odds ratio for transfusion reactions in the pre-storage group relative to the post-storage group was 0.57 (95% confidence interval [CI] 0.45–0.72, *P* < 0.01); the odds ratio after matching was 0.63 (95% CI 0.49–0.80, *P* < 0.01). A two-proportion *z*-test revealed pre-storage leukoreduction significantly decreases the symptoms of chills, fever, itching, urticaria, dyspnea, and hypertension as compared with those in post-storage leukoreduction. Pre-storage leukoreduced apheresis platelet significantly decreased febrile non-hemolytic transfusion reaction as compared with post-storage groups. This study suggests pre-storage leukoreduction apheresis platelet significantly decreases the transfusion reaction as compared with those in post-storage leukoreduction.

**Supplementary Information:**

The online version contains supplementary material available at 10.1007/s00277-024-05652-9.

## Introduction

Transfusion reactions are a major issue in transfusion medicine. Platelet transfusion can be accompanied by adverse reactions, and transfusion reactions are more frequent after the transfusion of platelets than after transfusion of other blood products [1, 2]. Platelet products can be divided into pooled donor platelet concentrates and single-donor apheresis platelets. Apheresis platelets have been widely applied in clinical practice because they provide several advantages over pooled donor concentrates, such as reduced exposure to donor antigens and lower risk of septic transfusion reactions [3]. Leukoreduction is another important strategy for preventing transfusion reactions. Removal of leukocytes from platelet products may reduce the risk of human leukocyte antigen (HLA) alloimmunization and platelet refractoriness [4, 5], reduce the severity and frequency of febrile non-hemolytic transfusion reactions (FNHTRs) [6–8], and lower the risk of cytomegalovirus transmission [9, 10]. For apheresis platelets, the leukoreduction process can be performed either before or after the platelets stored. Pre-storage leukoreduction removes leukocytes during the preparation of apheresis platelets, whereas post-storage leukoreduction removes leukocytes by filter from unmanipulated apheresis platelets during the transfusion process at bedside. Pre-storage leukoreduction is generally believed to be superior to post-storage leukoreduction in preventing transfusion reactions because the former method reduces cytokine release from leukocytes while the platelet product is in storage [11]. However, seldom studies have compared these two modalities of transfusion reaction prevention, especially for apheresis platelets. In Taiwan, the Taiwan Blood Service Foundation has been promoting pre-storage leukoreduction apheresis platelets since 2012. However, they continue to simultaneously provide both pre-storage leukoreduction apheresis platelets and non-leukoreduction apheresis platelets. Therefore, we conducted a retrospective study to compare the transfusion reactions between pre-storage and post-storage leukoreduced apheresis platelets by using a propensity-score-matching analysis.

## Methods

### Transfusion order review and enrollment criteria

We retrospectively reviewed all leukoreduced apheresis platelet transfusion orders for inpatients in Taipei Veterans General Hospital from August 2018 to December 2022. Data on each patient’s age, sex, blood type, and admission ward were obtained for analysis. Pooled platelet concentrate transfusions were excluded from this study. Additionally, transfusions for outpatients, pediatric patients less than 1-year-old, and patients admitted to the emergency department or intensive care unit were excluded. This study was approved by the Institutional Ethical Committee of Taipei Veterans General Hospital, and the study protocol was in agreement with the Helsinki Declaration of 1975, revised in 2008.

### Leukoreduction manufacturing process for apheresis platelets

Pre-storage leukoreduced apheresis platelets were prepared by the Taiwan Blood Service Foundation under the standard procedures. Platelets were collected using a leukocytapheresis device—Trima Accel^®^(Terumo BCT, USA), achieving a three-log reduction of leukocytes during apheresis [12, 13]. Post-storage leukoreduction was accomplished using a high-efficiency leukocyte filter—Haemonetics PXL8Y^®^(Haemonetics, USA) during the transfusion process at bedside [14]. Both pre-storage and post-storage leukoreduced apheresis platelets contained less than 5 × 10^6^ leukocytes per unit, as per the standards of the Association for the Advancement of Blood and Biotherapies [15].

### Transfusion reaction record and review

Patient vital signs, such as body temperature, heart rate, blood pressure, respiratory rate, and oxygen saturation, were checked before transfusion. Vital signs were monitored every 15 min until the transfusion was complete. Patients were subsequently monitored for 60 min after the platelet transfusion was completed. During and after transfusion, any onset of new symptoms that indicated a potential transfusion reaction, such as chills, fever, itching, face flushing, urticarial, nausea, dyspnea, stridor, hypotension, cold sweating, hypertension, and chest pain, were recorded. These patients were referred to transfusion medicine physicians for further investigation. Every transfusion reaction was independently reviewed by two transfusion medicine physicians. Each documented transfusion reaction, including the reaction categories and symptoms, were recorded in the patient’s chart.

### Statistical analysis

Each patient’s age, sex, blood type, admission ward, transfusion reaction, reaction category, and transfusion reaction-related symptoms are presented as number (*n*) and proportion (%). Logistic regression was used to determine the association between transfusion reactions and both pre-storage and post-storage leukoreduced apheresis platelets. A multivariate analysis adjusted for sex, age, and other clinical variables was performed to investigate the association between transfusion reactions and leukocyte-reduced apheresis platelets.

Propensity-score matching was performed to adjust for baseline differences between the pre-storage and post-storage groups [16]. Propensity score matching entails the selection of subsets from pre-storage leukoreduction group and post-storage leukoreduction group with similar covariate distributions, represented by propensity scores. This process aims to control for the confounding effects of covariates that might introduce bias into the estimated treatment effects. In 1-to-1 matching based on propensity scores, pre-storage subject are matched to post-storage subject, aligning the sample sizes between paired groups and ensuring similarity. Given the nature of this retrospective study, there were some inherent baseline differences (standardized mean difference > 0.1) in the original unmatched population between recipients of pre-storage and post-storage leukoreduced apheresis platelet transfusions. Propensity scores were generated through logistic regression by using pre-storage leukoreduction apheresis platelets as the dependent variable. Independent variables included age, sex, blood type, and admission ward. Propensity scores were obtained and applied to adjust for covariates in matched cases among pre-storage and post-storage leukoreduced apheresis platelet transfusions. Covariates were balanced between the matched groups and subsequently verified by the standardized mean difference. The conditional logistic regression was applied to determine the association between pre-storage leukoreduced apheresis platelets and transfusion reaction outcomes in paired groups after propensity-score matching. Logistic regression results are presented as odds ratio and 95% confidence interval (CI). Transfusion reaction categories and transfusion-related symptoms were compared using two proportion *z*-tests for pre-storage and post-storage leukoreduced apheresis platelets. Statistical tests were two-sided, and results were considered significant at *P* < 0.05. All analyses were performed using the R statistical software (The R Project for Statistical Computing, version 4.0.2).

## Results

### Characteristics and transfusion reactions of patients who received pre-storage or post-storage leukoreduced apheresis platelets

A total of 40,837 leukoreduced apheresis platelet transfusion orders were reviewed; 21,884 orders involved pre-storage leukoreduction and 18,953 orders involved post-storage leukoreduction. Detailed information relevant to the general characteristics of patients receiving pre-storage and post-storage leukoreduced apheresis platelets is shown in Table 1. The median age of the patients who received transfusions was 63 years (interquartile range: 51–73), and 55.8% were men. The majority of patients has blood type O, which is the most common blood type in Taiwan. Additionally, more than half of the transfusions were administered to cancer patients. Because this was a retrospective study, there were some inherent baseline differences (standardized mean difference > 0.1) between recipients of pre-storage and post-storage leukoreduced apheresis platelet transfusions: admission to neurology ward and age at 60 ~ 80 years old would receive more pre-storage leukoreduced apheresis platelet transfusion. We performed propensity-score matching to adjust for covariates between the pre-storage and post-storage cohorts and verified that by the standardized mean difference for each covariate less than 0.1. By propensity-score matching, a total of 18,314 matched cases were selected from original 18,953 post-storage and 21, 884 pre-storage. The histogram demonstrating propensity scores before and after matching is illustrated in Supplemental Fig. 1.

**Table 1:** Characteristics and transfusion reactions of patients receiving pre-storage or post-storage leukoreduced apheresis platelets

	Unmatched			Propensity-score matched		
	Post-storage (n = 18,953)	Pre-storage (n = 21,884)	SMD	Post-storage (n = 18,314)	Pre-storage (n = 18,314)	SMD
Sex, male, *n* (%)	10,510 (55.4)	12,290 (56.1)	0.014	10,182 (55.6)	9401 (51.3)	0.086
Age, no (%)						
≥ 80	2598 (13.7)	2691 (12.2)	0.042	2445 (13.4)	2439 (13.3)	0.001
60 ~ 80	7718 (40.7)	9897 (45.5)	0.091	7603 (41.6)	7156 (39.1)	0.050
40 ~ 60	5448 (28.8)	6173 (28.2)	0.012	5423 (29.7)	5704 (31.1)	0.003
< 40	3189 (16.8)	3123 (14.1)	0.071	2843 (15.3)	3015 (16.5)	0.026
Blood type, *n* (%)						
A	5353 (28.2)	6625 (30.3)	0.045	5290 (28.9)	5580 (30.4)	0.035
B	4722 (24.9)	4965 (22.7)	0.052	4514 (24.6)	3908 (21.4)	0.079
AB	1452 (7.7)	1291 (5.9)	0.040	1192 (6.5)	1267 (6.9)	0.027
O	7426 (39.2)	9003 (41.1)	0.070	7318 (40.0)	7559 (41.3)	0.016
Division ward, *n* (%)						
Medicine	4693 (24.8)	5011 (22.9)	0.044	4631 (25.3)	4020 (22.0)	0.079
Surgery	2039 (10.8)	3009 (13.7)	0.091	2005 (11.0)	2431 (13.3)	0.071
Neurology	303 (1.6)	867 (4.0)	0.144	266 (1.5)	262 (1.5)	0.002
Pediatrics	1352 (7.1)	1431 (6.5)	0.024	1202 (6.6)	1398 (7.6)	0.042
GYN/OBS	405 (2.1)	481 (2.2)	0.004	402 (2.2)	446 (2.4)	0.016
Orthopedics	44 (0.2)	200 (1)	0.090	43 (0.2)	47 (0.2)	0.004
Cancer	9997 (52.7)	10,776 (49.2)	0.070	9675 (52.8)	9,640 (52.7)	0.004
Geriatrics	51 (0.3)	28 (0.2)	0.032	21 (0.1)	24 (0.1)	0.005
Others	69 (0.4)	81 (0.3)	0.001	69 (0.3)	46 (0.2)	0.022
Transfusion reaction	174 (0.91)	116 (0.53)		166 (0.90)	106 (0.57)	

*SMD* standardized mean difference, *GYN/OBS* gynecology/obstetrics others include rehabilitation, hospice care, ophthalmology, psychology

### Transfusion reaction–related symptoms in patients who received pre-storage or post-storage leukoreduced apheresis platelets

**Fig. 1:**
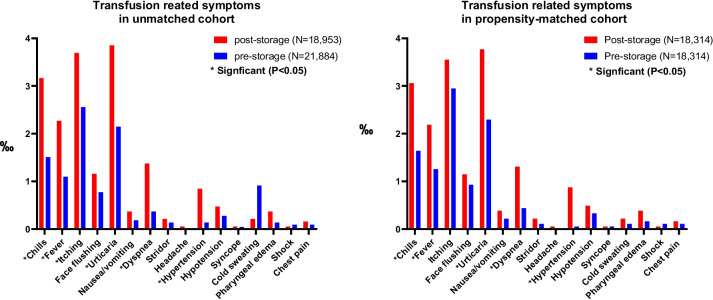
Transfusion-related symptoms in unmatched cohort and in propensity-matched cohort

Detailed information about transfusion reaction–related symptoms is shown in Table 2. A total of 211 and 347 transfusion reaction-related symptoms were reported in the pre-storage and post-storage leukoreduction groups, respectively. Itching was the most common symptom in pre-storage leukoreduction groups, while urticaria was the most frequent reported in post-storage. By two-proportion *z*-test analysis, the unmatched cohort showed that the pre-storage leukoreduction significantly decreased the symptoms of chills, fever, itching, urticaria, dyspnea, and hypertension as compared with those in post-storage leukoreduction. Other transfusion reaction symptoms, such as face flushing, nausea, vomiting, stridor, hypotension, syncope, cold sweating, pharyngeal edema, shock, and chest pain were not significantly different between pre-storage and post-storage leukoreduction groups. In propensity-score matched cohort, the pre-storage leukoreduction groups remained significantly lower symptoms of fever, chill, urticaria, dyspnea, and hypertension, but not itching. Figure 1 shows the incidence of transfusion reaction–related symptoms as well as comparison between pre-storage and post-storage leukoreduction apheresis platelet transfusion in unmatched and propensity-score matched cohort.

**Table 2:** Transfusion reaction related symptoms in patients receiving pre-storage and post-storage leukoreduced apheresis platelets

	Unmatched			Propensity-score matched		
	Post-storage (*n* = 18,953)	Pre-storage (*n* = 21,884)	*P* value*	Post-storage (*n* = 18,314)	Pre-storage (*n* = 18,314)	*P* value*
Chills, *n *(%)	60 (0.3165)	33 (0.1507)	< 0.001	56 (0.3057)	30 (0.1638)	0.004
Fever, *n* (%)	43 (0.2268)	24 (0.1096)	0.003	40 (0.2184)	23 (0.1255)	0.032
Itching, *n* (%)	70 (0.3693)	56 (0.2558)	0.039	65 (0.3549)	54 (0.2948)	0.312
Face flushing, *n *(%)	22 (0.1160)	17 (0.0776)	0.211	21 (0.1144)	17 (0.0928)	0.515
Urticaria, *n* (%)	73 (0.3851)	47 (0.2147)	0.001	69 (0.3767)	42 (0.2293)	0.010
Nausea and vomiting, *n* (%)	7 (0.0369)	4 (0.0182)	0.250	7 (0.0382)	4 (0.0218)	0.368
Dyspnea, *n* (%)	26 (0.1371)	8 (0.0365)	< 0.001	24 (0.1310)	8 (0.0436)	0.004
Stridor, *n* (%)	4 (0.0211)	3 (0.0137)	0.568	4 (0.0218)	2 (0.0109)	0.412
Headache, *n* (%)	1 (0.0052)	0 (0.000)	-	1 (0.0054)	0 (0.000)	-
Hypertension, *n* (%)	16 (0.0844)	3 (0.0137)	< 0.001	16 (0.0873)	1 (0.0054)	< 0.001
Hypotension, *n* (%)	9 (0.0474)	6 (0.0274)	0.289	9 (0.0491)	6 (0.0327)	0.441
Syncope, *n* (%)	1 (0.0052)	1 (0.0045)	0.920	1 (0.0054)	1 (0.0054)	> 0.999
Cold sweating, *n* (%)	4 (0.0211)	2 (0.091)	0.322	4 (0.2184)	2 (0.0109)	0.412
Pharyngeal edema, *n* (%)	7 (0.0369)	3 (0.0137)	0.133	7 (0.0382)	3 (0.0163)	0.204
Shock, *n* (%)	1 (0.0052)	2 (0.0091)	0.652	1 (0.0054)	2 (0.0109)	0.561
Chest pain, *n *(%)	3 (0.0158)	2 (0.0091)	0.541	3 (0.0163)	2 (0.0109)	0.652
Total, *n* (%)	347 (1.8308)	211 (0.9641)	< 0.001	328 (1.7909)	197 (1.0756)	< 0.001

^*^Estimated by two proportion *z*-test

### Transfusion reaction comparison between pre-storage and post-storage leukoreduced apheresis platelets

Statistical results are shown in Table 3. A total of 116 transfusion reactions were reported among 21,884 pre-storage leukoreduced apheresis platelet transfusions, and 174 reactions were reported among 18,953 post-storage leukoreduced transfusions. The statistical analyses revealed the pre-storage leukoreduced apheresis platelet significantly decrease the transfusion reaction as compared the post-storage groups in all statistical analysis, including crude rate, multivariate analysis adjusted for sex, age, and other covariates, as well as conditional logistic regression after propensity-score matching.

**Table 3:** Transfusion reaction comparison in pre-storage and post-storage leukoreduced apheresis platelets

Leukoreduction apheresis platelets	Transfusion reaction/number at risk (%)
Pre-storage	116/21,884 (0.53)
Post-storage	174/18,953 (0.91)
Analysis for pre-storage versus post-storage	Odds ratio (95%CI), *P*-value
Crude analysis	0.575 (0.454–0.728), < 0.01
Multivariable analysis	0.572 (0.450–0.725), < 0.01
Propensity score analysis	0.631 (0.493–0.808), < 0.01

*CI* confidence interval

### Transfusion reaction category comparison in pre-storage and post-storage leukoreduced apheresis platelets

The transfusion reaction categories are shown in Table 4. The two proportion *z*-test analysis revealed the pre-storage leukoreduced apheresis platelet significantly decreased FNHTR as compared with post-storage groups. These results remained significant in propensity-score matched cohort.

**Table 4:** Transfusion reaction categories in patients receiving pre-storage and post-storage leukoreduced apheresis platelets

	Unmatched			Propensity-score matched		
	Post-storage (*n* = 18,953)	Pre-storage (*n* = 21,884)	*P* value*	Post-storage (*n* = 18,314)	Pre-storage (*n* = 18,314)	*P* value*
Febrile non-hemolytic transfusion reaction, *n *(%)	64 (0.3376)	41 (0.1873)	0.002	62 (0.3385)	37 (0.2020)	0.011
Transfusion associated dyspnea, *n *(%)	2 (0.0105)	1 (0.0045)	0.483	2 (0.0109)	1 (0.0054)	0.561
Hypotension, *n *(%)	3 (0.0158)	0 (0.0000)	-	3 (0.0163)	0	-
Transfusion associated circulatory overload, *n *(%)	5 (0.0263)	1 (0.0045)	0.073	5 (0.0273)	1 (0.0054)	0.103

^*^Estimated by two proportion *z*-test

## Discussion

Removal of leukocytes from blood products has been shown to reduce HLA alloimmunization, FNHTR, and platelet refractoriness. A randomized controlled clinical trial assigned eligible patients to receive either un-manipulated or leukoreduced blood products [17]. This study revealed no significant difference in in-hospital mortality, hospital length of stay, and total hospital cost between un-manipulated and leukoreduced blood products; however, patients who received leukoreduced blood products had a lower incidence of febrile reactions [17]. Most of the studies mentioned above involved pooled platelet concentrates. However, single-donor apheresis platelet transfusion has several advantages over pooled platelet concentrate transfusions, such as lower donor HLA exposure, less inventory and pooling, easier HLA matching, and reduced risk of septic transfusion reactions. Therefore, apheresis platelets have become the preferred platelet product, but few studies have focused on the effects of pre-storage versus post-storage leukoreduction in platelet apheresis. A recent study observed that FNHTR incidence was 0.07% in leukoreduced single donor apheresis platelets, 0.16% in pre-storage leukoreduced pooled platelets, 0.30% in post-storage leukoreduced pooled platelets, and 0.20% in non-leukoreduced pooled platelets [8]. These results imply that single-donor apheresis platelets may be superior to pooled platelets, regardless of the application or timing of leukoreduction in the pooled platelet products. However, it is not clear if pre-storage leukoreduction is superior to post-storage leukoreduction in apheresis platelet products. Furthermore, Mishima et al. [18] found that compared with bedside leukoreduction, universal leukoreduction was associated with a significantly higher rate of antibody detection (8.7% vs. 5.4%) and platelet transfusion refractoriness (7.3% vs. 3.2%). Therefore, it is worth investigating whether pre-storage leukoreduction platelet apheresis prevent more transfusion reaction. Our study exactly provides the evidence of pre-storage leukoreduced platelet apheresis significantly deceasing the transfusion reaction as compared with post-storage in a relatively large cohort research.

FNHTR is a very common transfusion reaction, and substantial research has revealed several underlying mechanisms of this reaction. Donor-derived leukocytes may interact with the recipient’s anti-leukocyte antibodies as a result of the antigen–antibody complement reaction and cytokine release [6]. This interaction can be mitigated by leukocyte removal through either pre-storage or post-storage leukoreduction. Leukocytes also release pyrogenic cytokines, such as interleukin-6, interleukin-8, tumor necrosis factor-alpha, and interleukin-1, which are believed to contribute to transfusion reactions. One advantage of pre-storage leukoreduction over post-storage leukoreduction is the minimization of cytokine accumulation during storage. This is a general reason why pre-storage leukoreduction is superior to post-storage leukoreduction [19]. In addition to cytokines released from leukocytes, the secretion of alpha granules and granular platelet chemokines can also induce transfusion reactions. The platelet-derived chemokines CCL3, CCL5, and CXCL4 can cause FNTHR [20, 21]. Neither pre-storage nor post-storage leukoreduction can filter out these platelet-derived chemokines and granules. This may explain although pre-storage leukoreduction platelet apheresis could alleviate the transfusion reaction more efficiently, it could not eliminate all the transfusion reaction.

In addition to decrease transfusion reaction, there are several well-known advantages and disadvantages of pre-storage leukoreduction. For example, a study found that pre-storage leukoreduction reduced not only the release of inflammatory cytokines from leukocytes but also the risk of virus transmission due to the release of intracellular organisms from leukocytes during storage [15]. Additionally, it is easier to control the quality of leukocyte removal in a donor center rather than at bedside. The major disadvantages of pre-storage or universal leukoreduction are the cost involved and the inherent issue of inventory management, as it is difficult to estimate the demand for leukoreduced platelets at the time of blood product preparation [22]. However, post-storage or bedside leukoreduction could be selectively applied to certain populations for which leukoreduced blood products are recommended, which could mitigate cost issues. In our study, the crude rate of transfusion reactions for pre-storage and post-storage leukoreduction groups was 0.53% and 0.91%, respectively. The number needed to treat to avoid one transfusion reaction with pre-storage rather than post-storage leukoreduced platelets was 263. This implies that for every 263 transfusions, one transfusion reaction could be prevented by opting for pre-storage leukoreduction platelet transfusion over post-storage leukoreduction. A cost-effective analysis should be considered when implementing pre-storage or post-storage apheresis platelet transfusions.

Our study had several inherent limitations. First, this was a retrospective study rather than a randomized controlled trial, so the baseline characteristics of the patients differed between the pre-storage and post-storage groups. Therefore, we performed propensity-score matching for the cohort to minimize potential selection bias. Second, only inpatients were included in the cohort to facilitate the follow-up and monitoring of transfusion reactions. Although our results may be generalizable to the majority of inpatients, this cohort is not representative of the entire transfusion candidate population. Third, our study focused on acute transfusion reactions but did not compare alloimmunization or platelet refractoriness between the pre-storage and post-storage groups. Finally, blood product selection should be dependent on each patient’s characteristics. A large-scale, randomized controlled clinical trial is necessary to identify the characteristics of patients who may benefit from pre-storage leukoreduced apheresis platelet transfusions.

## Conclusion

In conclusion, this study suggests pre-storage leukoreduction apheresis platelet significantly decreases the transfusion reaction as compared with those in post-storage leukoreduction, especially in FNHTR. The crude rate of transfusion reactions for pre-storage and post-storage leukoreduction groups was 0.53% and 0.91%, respectively. The number needed to treat to avoid one transfusion reaction with pre-storage rather than post-storage leukoreduced platelets was 263. This study suggests that pre-storage leukoreduction apheresis platelet is an optimal choice in the supply of blood products.

## Funding

This study is partially supported by the grants from Taipei Veterans General Hospital-National Yang-Ming University Excellent Physician Scientist Cultivation Program, No.112-V-A-072, Szu-Yuan Research Foundation of Internal Medicine.

### Supplementary Information

Below is the link to the electronic supplementary material.

Supplementary file1 (PDF 5 KB)

## Declarations

This study was approved by the Institutional Ethical Committee of Taipei Veterans General Hospital, and the study protocol was in agreement with the Helsinki Declaration of 1975, revised in 2008.
