# Supplementary figures and images for: Comparing transfusion reactions between pre-storage and post-storage leukoreduced apheresis platelets: an analysis using propensity score matching
Source: Ann Hematol. 2024 Feb 23;103(4):1389–96. doi: 10.1007/s00277-024-05652-9 (PMC10940477; doi:10.1007/s00277-024-05652-9)

**Raw Treated**

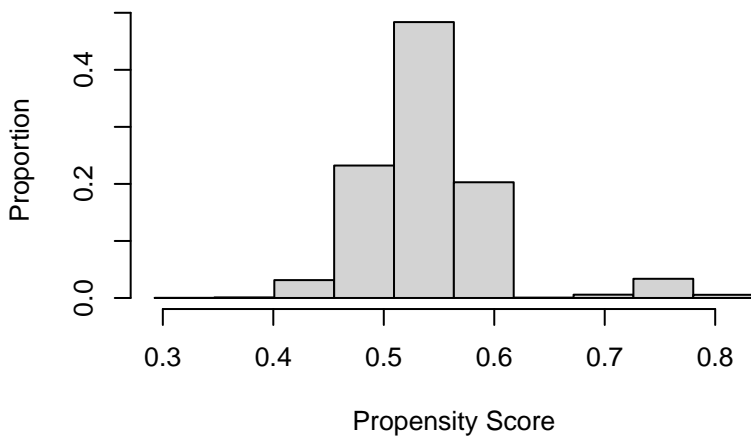

**Matched Treated**

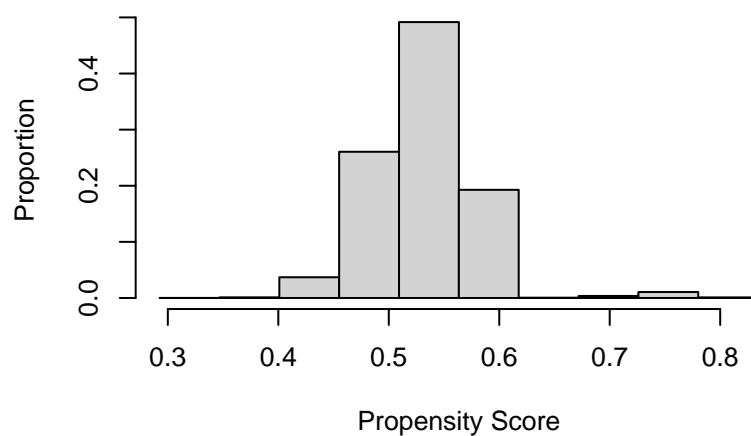

**Raw Control**

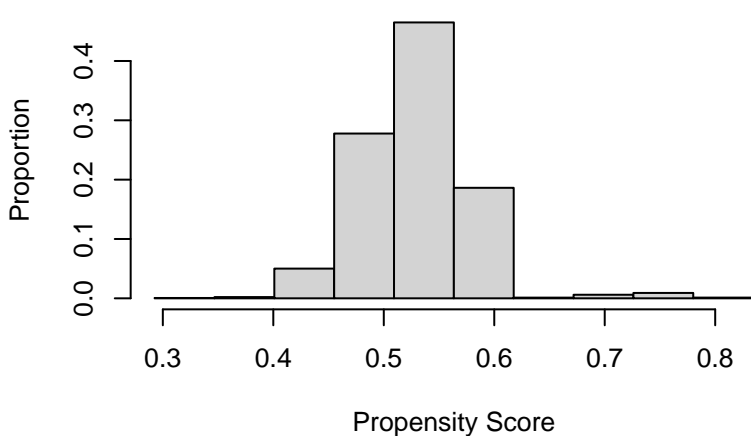

**Matched Control**

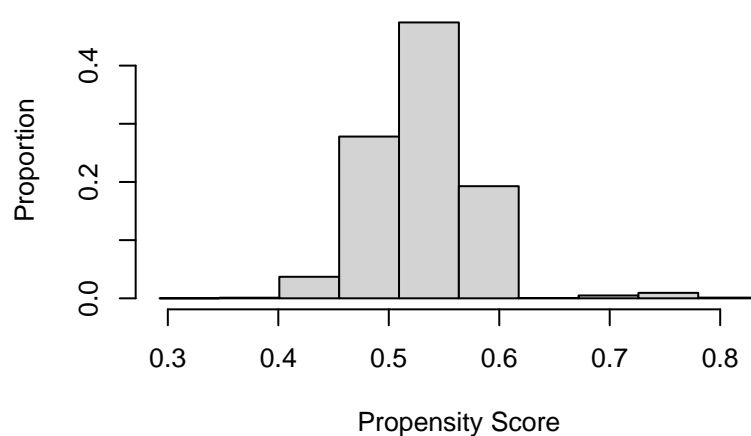

Supplement: Supplementary file 1 — Supplementary file1 (PDF 5 KB) [file 277_2024_5652_MOESM1_ESM.pdf]
